# Patient delay in the diagnosis of tuberculosis in Ethiopia: a systematic review and meta-analysis

**DOI:** 10.1186/s12879-020-05524-3

**Published:** 2020-10-27

**Authors:** Muluneh Alene, Moges Agazhe Assemie, Leltework Yismaw, Getnet Gedif, Daniel Bekele Ketema, Wodaje Gietaneh, Tadele Demilew Chekol

**Affiliations:** 1 Department of Public Health, Debre Markos University, Debre Markos, Ethiopia; 2 School of Medicine, Debre Markos University, Debre Markos, Ethiopia

**Keywords:** Delay, Median, Tuberculosis, Ethiopia, Meta-analysis

## Abstract

**Background:**

Delay in the diagnosis of Tuberculosis (TB) remains a major challenge against achieving effective TB prevention and control. Though a number of studies with inconsistent findings were conducted in Ethiopia; unavailability of a nationwide study determining the median time of patient delays to TB diagnosis is an important research gap. Therefore, this study aimed to determine the pooled median time of the patient delay to TB diagnosis and its determinants in Ethiopia.

**Methods:**

We followed PRISMA checklist to present this study. We searched from Google Scholar, PubMed, Science Direct, Web of Science, CINAHL, and Cochrane Library databases for studies. The comprehensive search for relevant studies was done by two of the authors (MA and LY) up to the 10th of October 2019. Risk of bias was assessed using the Newcastle-Ottawa scale adapted for observational studies. Data were pooled and a random effect meta-analysis model was fitted to provide the overall median time of patient delay and its determinants in Ethiopia. Furthermore, subgroup analyses were conducted to investigate how the median time of patient delay varies across different groups of studies.

**Results:**

Twenty-four studies that satisfied the eligibility criteria were included. Our meta-analysis showed that the median time of the patient delay was 24.6 (95%CI: 20.8–28.4) days. Living in rural area (OR: 2.19, 95%CI: 1.51–3.18), and poor knowledge about TB (OR: 2.85, 95%CI: 1.49–5.47) were more likely to lead to prolonged delay. Patients who consult non-formal health providers (OR: 5.08, 95%CI: 1.56–16.59) had a prolonged delay in the diagnosis of TB. Moreover, the narrative review of this study showed that age, educational level, financial burden and distance travel to reach the nearest health facility were significantly associated with a patient delay in the diagnosis of TB.

**Conclusions:**

In conclusion, patients are delayed more-than three weeks in the diagnosis of TB. Lack of awareness about TB, consulting non-formal health provider, and being in the rural area had increased patient delay to TB diagnosis. Increasing public awareness about TB, particularly in rural and disadvantaged areas could help to early diagnosis of TB.

**Supplementary information:**

**Supplementary information** accompanies this paper at 10.1186/s12879-020-05524-3.

## Background

In 2017, an estimated 1.6 million people died due to treatable and curable disease called tuberculosis (TB) [1]. It is one of the top ten causes of death worldwide, and over 95% of cases and deaths are occurring in developing countries [2]. Though Ethiopia has achieved the reduction of TB incidence by halve, the decline of its incidence and prevalence rates has been comparatively slow [3].

In developing countries, delay in the diagnosis of TB remains a major challenge against effective management of the disease. Previous report showed that 42% patients are delayed to TB treatment in low-and middle-income countries [4]. Delay in seeking care worsen the burden of TB and the cost of care for patients, families and the overall public health system [5]. Patient-level and system-level barriers including limited knowledge, attitude, belief regarding TB, economic burdens, centralization of services, health system delays, and geographical access to healthcare influenced timely TB treatment initiation and compliances [6, 7].

The average time of patient delay in the diagnosis of TB and its determinants were reported by a number of studies [8–22]. However, the reported average time and determinant factors in these fragmented studies vary depending on the characteristics of study participants, the type of design employed and the variables analyzed. Having conclusive evidence by combining the existing studies is significant to have quality evidence on the national TB prevention and control program. Thus, this systematic review and meta-analysis aimed to determine the pooled median time of delay in the diagnosis of TB patients and to identify its determinants. This finding will help health-policy makers and other concerned body to apply efficient interventions, and to improve the health care seeking behavior of TB patients.

## Methods

### Study design and setting

A study that aimed to estimate the pooled median time of patient delay in the diagnosis of TB and to identify its determinants was conducted in Ethiopia. The country is subdivided into ten national regional states: namely Amhara, Tigray, Oromia, Afar, Benishangul-Gumuz, Somali, Southern Nations Nationalities and People Region (SNNPR), Sidama, Gambella, Harari, and two city administrative states (Dire Dawa city council and Addis Ababa city administration).

### Eligibility criteria

#### Criteria for including studies

##### Study design

All health facility-based cross-sectional studies reporting a patient delay in the diagnosis of pulmonary TB.

##### Study setting

All studies reporting a patient delay in the diagnosis of pulmonary TB conducted in Ethiopia.

##### Participants

All smear positive and negative pulmonary TB patients.

##### Outcome

Studies which report the median time of patient delay for TB diagnosis.

##### Articles

Published and unpublished studies.

#### Criteria for excluding studies

We excluded articles that were not fully accessed after at least two email contacts of the principal investigator.

#### Searching for studies

We followed the Preferred Reporting Items for Systematic Reviews and Meta-Analysis (PRISMA) checklist to present this study [23]. The comprehensive search for potential studies was conducted by two of the authors (MA and LY) up to the 10th of October 2019 without limitation to the year of publication. Two experienced review authors (MA and LY) searched from Google Scholar, Science Direct, PubMed, Cochrane library, Web of Science, and CINAHL databases for studies. The search from the above stated databases was performed using the following keywords: “patient delay” OR “total delay” AND “determinants” OR “factors” AND “Ethiopia”.

### Outcome measures and data extraction

Patient delay in the diagnosis of TB is the outcome variable of this study. It is the time from symptom onset to first consultation of the formal health system. Symptom onset referred to the time at which the first symptom such as chronic cough, fever, weight loss and night sweats of the illness began. Furthermore, the patient delay was categorized using a median cutoff point of 30 days. That is, greater than 30 days was taken as a prolonged patient delay. All relevant information from the included studies were extracted independently by two (MA and MAA) of authors after data extraction checklist development. It includes; the last name of the first author, publication year, the region of the study conducted, data collection period, study population, sample size, response rate, and median time of patient delay [24].

### Quality assessment tool

Two reviewers (MA and DBK) assessed the quality of articles before inclusion to maintain methodological validity. The Newcastle-Ottawa Scale adapted for cross-sectional studies was used to assess the risk of bias [25]. The tool organized in three sections with a maximum of ten score. The first section scored a maximum of five stars and focuses on the representativeness of the sample. The second section concerned on how the confounding variables controlled with a maximum of two stars. The third section is focused with the outcomes and statistical analysis of study with a possibility of three stars to be gained.

Finally, the average score provided by two reviewers was taken. Articles scored seven and above were considered as achieving high quality. This cut-off point was considered after referring previous literature [26].

### Data processing and analysis

All relevant information was extracted using Microsoft excel software. R statistical software employed for meta-analysis. We used the quintile estimation method to estimate the overall medians time among the included studies. This approach had considerably lower absolute percent error than pooled means estimated via transformation-based approaches [27]. Bowley’s coefficient of skewness (SKb) was used to measure the skewness of data and if the mean *SKb* greater than 0.1 median-based approaches are suggested.

We also conducted a meta-analysis on factors of patients delay in the diagnosis of TB. Since most of the included studies reported that the median patient delay was 30 days, we used it as a cut of point to dichotomize patient’s delay. Moreover, subgroup analyses were conducted to investigate how the median of patient delay in the diagnosis of TB varies across different subgroups of the studies. The region of study conducted, year of publication, and the number of study participants incorporated are subgroups studied in this review.

## Results

### Search results

**Fig. 1:**
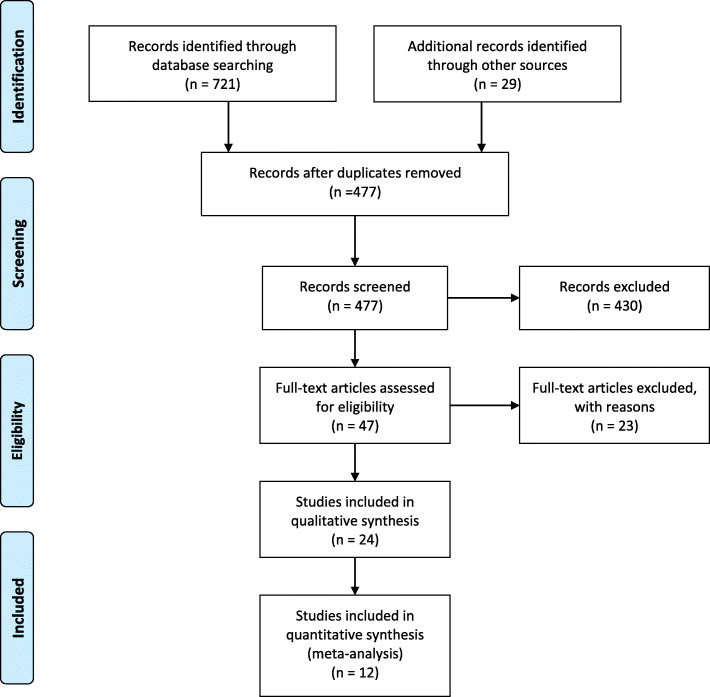
Flow chart diagram describing the selection of studies included in the systematic review and meta-analysis of patient delay in diagnosis of tuberculosis in Ethiopia

Figure 1, shows the number of literature searched, study selection, and the number of studies included. A total of 750 articles were identified during our search, and then 477 articles were excluded due to duplication. Twenty-four studies that satisfied the eligibility criteria were included in this systematic review and meta-analysis.

### Description of the included studies

In this study, we included a total of twenty-four articles (Table 1). All the included studies were institutional-based cross-sectional. Administratively, nine (41.7%) studies were conducted in the Amhara region, four studies in Oromia region, three studies in South Nations Nationalities and Peoples Regions (SNNPR), and two studies in Addis Ababa. The publication year of the included studies was between 2002 and 2019. The number of study participants among included studies ranged from 105 to 735. The smallest reported median time of the patient delay in the diagnosis of TB was 17 days [28], while the highest was 63 days [8]. Two-third (66.67%) of the included studies had a high quality (Table S1).

**Table 1:** Characteristics of the included studies conducted in Ethiopia on patients delay in Tuberculosis diagnosis

First author (publication year)	Region	Data collection period	Study population	Sample size	Prolonged delay classification (days)	Response rate	Median (IQR)	Prolonged delay (%)
Adenager et al. (2017) [28]	Addis Ababa	April to June 2012	Both SP and SN PTB patients	422	> 21	99.76%	17 (9–33)	42.1%
Alema et al. (2019) [16]	Tigray	Nov. 1, 2015 to Jan.30, 2016	new PTB patients	422	> 30		30 (21–60)	
Asefa et al. (2014) [29]	SNNPR	June to December 2012	SPPTB patients	328	> 30	NR	30 (20.2–60)	NR
Asres et al. (2017) [17]	Amhara	April 8 to July 7, 2013	All newly diagnosed TB patients	605	> 30	100%	45 (3–425)	53.4%
Asres et al. (2019) [18]	SNNPR	January to December 2015	all new SP, SN and EPTB cases	735	> 25	NR	25 (15–36)	NR
Belay et al. (2012) [30]	Afar	September 2009 and March 2010	TB patients	216	> 20	78%	20 (8–60)	76%
Bogale et al. (2017) [31]	Amhara	February to May, 2016	PTB cases	296	NR	NR	Mean, 33.9(sd = 14)	NR
Demissie et al. (2002) [32]	Addis Abeba	August 1 to December 311,998	Newly PTB patients	700	> 30	NR	60	NR
Fuge et al. (2018) [9]	SNNRP	May and September, 2016	TB patients	398	> 21	99.3	30 (5–120)*	58.2%
Gebeyehu et al. (2014) [10]	Amhara	January to April, 2013	SP, SN and EPTB	376	> 21	NR	SP27(10–59) SN 30 (9–65)	NR
Gebreegziabher et al. (2016) [19]	Amhara	Oct 2013 to Oct 2014.	All new PTB patients	706	> 30	NR	18 (8–34)	NR
Getnet et al. (2019) [11]	Somali	between December 2017 and October 2018	All PTB patients	442	> 30	NR	For cases:50 (40–72) For controls: 20 (14–25)	48.87%
Hussen et al. (2012) [8]	Oromiya	February to March 2011	All pulmonary TB patients	129	> 14	96%	63 (14–896)* * = range	NR
Mekonnen et al. (2014) [22]	Amhara	10 March – 08 May 2012	TB patients	315	NR	NR	30 (3–270)* * = range	52.4%
Mesfin et al. (2005) [33]	Tigray	NR	TB patients	237	> 21	NR	SPPTB:90 SNPTB:60 EXTB:90	NR
Seid et al. (2018) [34]	Amhara	April1, 2016 to January 30, 2017	TB patients	382	> 30	NR	30 (15–60)	41%
Shiferaw et al. (2019) [35]	Amhara	01 to 30 December 2017	All TB patients	170	> 21	95.3%	Mean: 53.2 (± 8.54).	59.9%
Tsegaye et al. (2016) [12]	Amhara	July 1 to September 30, 2013	All PTB patients	528	> 30	99.1%	36 (36)	62.3%
Wondimu et al. (2007) [13]	Oromiya	January 11, 2006 to April 11, 2006	All PTB patients	201	NR	99.5%	28	NR
Yarlagadda et al. (2018) [36]	Oromiya	February 9 to 20/2015	All PTB patients	105	> 21	NR	91 (mean)	58.09%
Yimer et al. (2005) [37]	Amhara	September 1, 2003 and December 31, 2003	new smear positive PTB patients	384	> 30	NR	30 (15–90)	NR
Yimer et al. (2014) [14]	Amhara	January to August 2010	All PTB patients	201	> 30	NR	21 (7–60]	68.7%
Yirgu et al. (2017) [20]	Oromiya	June to July 2014	All PTB patients	358	> 14	100%	15 (5–30)	
Zeleke et al. (2014) [15]	SNNR	March 2013 to February 2014	SPPTB patients	221	> 35	98.6%	35	NR

*SNNRP* Southern nations, nationalities, and peoples’ region, *NR* Not Report, *SPPTB* Smear positive pulmonary tuberculosis, *SN* smear negative, *EPTB* extra pulmonary tuberculosis

* = *P* < 0.05 (The 95%CI does not include one)

### Magnitude of patient delay in the diagnosis of TB

To pool the median time of the patient delay, we included studies that report the following summary measures; sample size, median, first quartile and third quartile. Finally, a total of twelve primary studies were included. The pooled median time of patient delay in the diagnosis of TB was 24.6 (95%CI: 20.8–28.4) days.

Subgroup analysis was undertaken by the region of study conducted, publication year, sample size (below median versus above median), and the quality of the study.

The highest median time of the patient delay to TB diagnosis was reported in SNNPR (27.2 (95%CI: 22.3–32.0)) days, while the smallest median time to TB diagnosis was observed in the Oromiya region (15.0 (95%CI: 12.7–17.3)) days. Consequently, the overall median time of patient delay to TB diagnosis was 25.8 (95%CI: 21.6–29.9) days among articles published before 2015, while it was 24.3 (95%CI: 18.0–30.5) days among articles published after 2015. In addition, the overall median time of the patient delay to TB diagnosis was 23.8 (95%CI: 18.7–28.9) days among articles included less-than 384 study participants, while it was 25.9 (95%CI: 19.3–32.5) days among articles included 384 and above study participants (Table 2).

**Table 2:** Subgroup analysis of studies included in meta-analysis on patient delay in diagnosis of pulmonary tuberculosis in Ethiopia (*n* = 12)

Subgroup	Number of included studies	Random effects (95%CI)	Test of heterogeneity (I^**2**^)	p- value
**By region**				
Amhara	6	26.8 (20.9–32.6)	90.6%	< 0.001
SNNPR	2	27.2 (22.3–32.0)	81.9%	< 0.001
Addis Ababa	1	17.0 (15.0–18.9)	_	
Oromiya	1	15.0 (12.7–17.3)	_	
Afar	1	20.0 (14.6–25.4)	_	
Tigray	1	30.0 (26.6–33.4)	–	
**By publication year**				
Before 2015	5	25.8 (21.6–29.9)	71.5%	< 0.001
After 2015	7	24.3 (18.0–30.5)	97.8%	< 0.001
**By sample size**				
< 384	6	23.8 (18.7–28.9)	90.2%	< 0.001
≥ 384	6	25.9 (19.3–32.5)	97.5%	< 0.001
Overall		24.6 (20.8–28.4)	94.9%	

### Determinants of patient delay in the diagnosis of TB

**Fig. 2:**
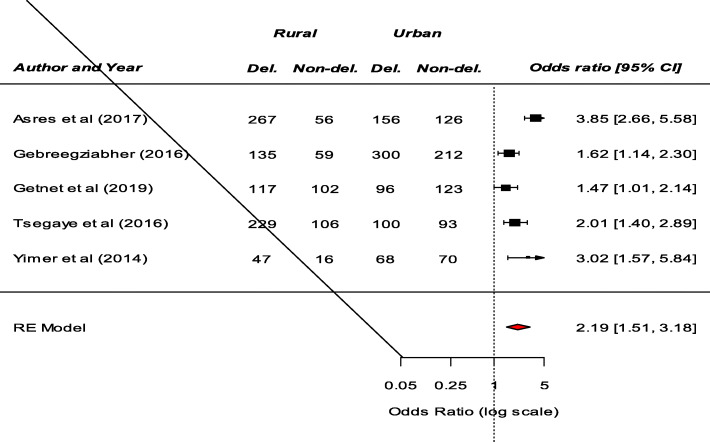
The pooled odds ratio of the association between residence and patient delay in the diagnosis of TB in Ethiopia

**Fig. 3:**
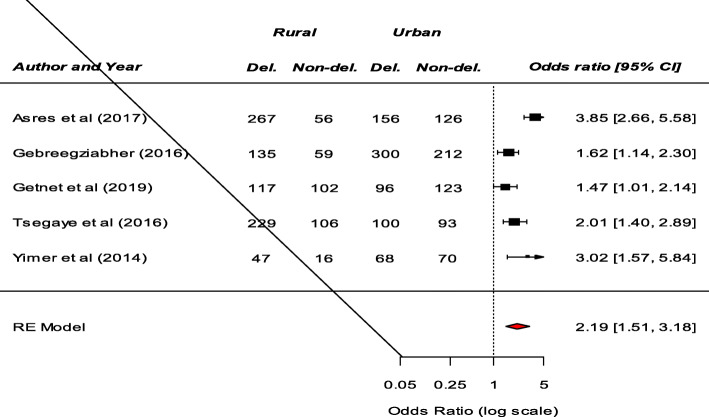
The pooled odds ratio of the association between knowledge and patient delay in the diagnosis of TB in Ethiopia

**Fig. 4:**
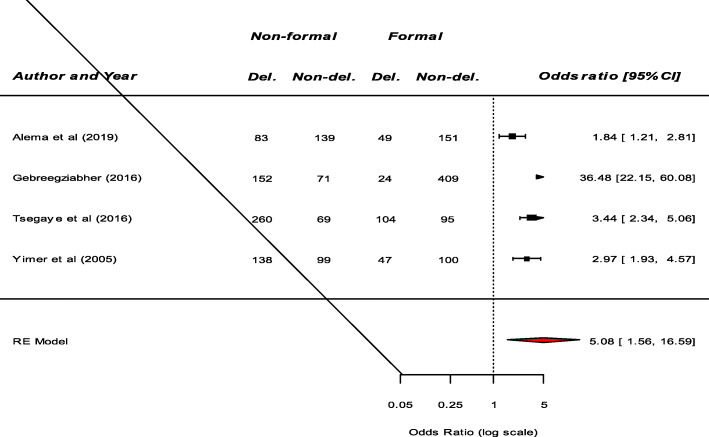
The pooled odds ratio of the association between first action to illness and patient delay in the diagnosis of TB in Ethiopia

Significant determinants of patient delay in the diagnosis of TB among the included studies were presented in (Table S2). Fourteen studies examined the association between residence and patient delay in the diagnosis of TB. Consequently, a significant association between residence and patient delay was reported by eight studies [8–15]. Patients living in the rural area had prolonged delay compared to patients living in urban area. Our meta-analysis also revealed that patients from rural area were more likely to have a prolonged delay (OR: 2.19, 95%CI: 1.51–3.18) (Fig. 2). Among nine studies that examine the association between knowledge about TB and patients delay, a significant result was reported by six studies [9, 11, 12, 16–18]. Our pooled analysis showed that patient delay is more likely among patients who had poor knowledge about TB (OR: 2.85, 95%CI: 1.49–5.47) (Fig. 3). Moreover, this meta-analysis showed that seeking treatment from non-formal providers was a significant risk factor for longer health care-seeking delay of TB (OR: 5.08, 95%CI: 1.56–16.59) (Fig. 4).

A significant association between the age of patient and prolonged delay was reported by five studies [17–20, 37]. Younger age group had a shorter time of TB diagnosis. In addition, a significant association between the educational level of a patient and patient delay was reported by five studies [8, 10, 13, 21, 22]. Consequently, as the educational level of the patient increases the likelihood of patient delay to TB diagnosis will be decreased. The other important variable which shows significant association to patient delay was time to reach the nearest health facility [8, 9, 11, 18, 22, 28, 37]. As the time elapses to reach the formal health provider increase, patients are more likely to have a prolonged delay. From the included studies, three studies reported that financial problem [9, 13, 16] was associated with patient delay to diagnosis. Participants who had better economic status are less likely to have prolonged delays.

## Discussion

This study was conducted to determine the pooled median time of patient delay in the diagnosis TB and to identify its determinants. The median time of patient delay among the included studies ranged from 17 to 63 days. The meta-analysis showed that the overall median time of patient delay in Ethiopia was 24.6 (95%CI: 20.8–28.4) days. This result is comparable with other studies conducted in Tanzania, Uganda and India [38–40]. On the other hand, the median time of patient delay obtained in this study was shorter than previous studies conducted Mozambique, Angola and Ghana [41–43], and higher than studies conducted in Cameroon and China [44, 45]. The possible explanation for this inconsistency might be differences in the socio-economic and demographic characteristics of study participants [46, 47]. The subgroup analysis by year of publication showed that the median time of patient delay among articles published after 2015 was shorter than articles published prior to 2015. This could be due to changes in policy and guidelines. In addition, a longer median time of patient delay was observed among studies with larger sample size (≥384) than studies with smaller sample size (< 384).

The pooled result of this study revealed that patients living in rural area were more likely to have a prolonged delay. This finding is consistent with a study conducted in Nigeria [48]. This might be due to the shortage of formal health providers in rural area of Ethiopia [49]. Long distance travelling discourages tuberculosis treatment initiation [7]. In addition, previous reports revealed that in rural areas, the first action to illness is consulting traditional healers [50]. Furthermore, a study conducted in the rural area of Nigeria showed that about 84% of TB patients first consulting a non-formal health provider [48].

Our pooled analysis showed that patient delay is more likely among patients who had poor knowledge about TB. This result is comparable with a study conducted in Mozambique, Bangladesh, and India [42, 51, 52]. The reason for this result might be patients who have poor knowledge of TB believe that TB is acquired from evil and they seek traditional healers or religious leaders to be freed from evil spirits [53]. This meta-analysis showed that seeking treatment from non-formal providers was a significant risk factor for longer patient delay of TB. A comparable result was obtained from studies conducted in low and middle-income countries, Mozambique, Uganda and Tanzania [4, 40, 42, 54]. This might be due to low knowledge tuberculosis [47]. Number and type of non-formal provider first consulted were the most important risk factors for delay [38].

Due to inconsistent classification of age, we were unable to show the pooled effects. In two studies, patient delay was longer among study participants whose age are forty five and above [19, 37]. The possible reason for this result might be older patients are dependent on others help and may not early seek health care. A study conducted in southern Ethiopia indicated that the health care seeking behaviors among elderly people is generally low [55].

Our narrative reviews also showed that the educational level of respondents was associated with patient delay in the diagnosis of TB. Consequently, as the educational level of patient increases the likelihood of patient delay to TB diagnosis will decreases, as found in few studies [8, 10, 13, 21]. This result is supported by other studies conducted in South Africa [46]. The possible reason for this result might be study participants who had higher educational levels were more likely to be aware of TB than those who had no education [56].

In addition, as the time elapse to reach the formal health provider increase patients are more-likely to have prolonged delay [8, 9, 11, 18, 28, 37]. This result is consistent with a study conducted in Angola and Uganda [41, 57]. A systematic review and meta-analysis study conducted in Asia revealed that long travel time to the nearest healthcare provider led to longer patient delays [58].

Participants who had better economic status are less-likely to have prolonged delay [9, 13, 16]. The possible explanation for this result might be patients suffered high economic losses prior to diagnosis [59]. The total median cost incurred from first consultation to diagnosis was $27 per patient in Ethiopia [5].

### Limitations

This systematic review and meta-analysis was not without limitations. Firstly, the review was limited to only articles published in the English language. Secondly, all of the included studies were institution based cross-sectional studies, which limits assessment of the cause-effect relationships. Thirdly, there is sizable in-consistency across the included studies. The observed heterogeneity might be described by the quality of the studies.

## Conclusions

In conclusion, patients are delayed more-than three weeks in the diagnosis of TB. Lack of awareness about TB, consulting non-formal health provider, and living in the rural area had increased patient delay to TB diagnosis. Increasing public awareness about TB and active case finding, particularly in rural and disadvantaged areas could help to early diagnosis of TB.

## Supplementary information

**Additional file 1: Table S1.** Assessing the risk of bias for the included studies.

**Additional file 2: Table S2.** Significant determinants of unintended pregnancy reported from each study.

## Data Availability

All data are available in the manuscript.

## Abbreviations

TB: Tuberculosis
PRISMA: Preferred Reporting Items for Systematic Reviews and Meta-Analysis
