# Supplementary material for: Patient delay in the diagnosis of tuberculosis in Ethiopia: a systematic review and meta-analysis
Source: BMC Infect Dis. 2020 Oct 27;20:797. doi: 10.1186/s12879-020-05524-3 (PMC7590610; doi:10.1186/s12879-020-05524-3)
Supplement: Supplementary file 2 — Additional file 2: Table S2. Significant determinants of unintended pregnancy reported from each study. [file 12879_2020_5524_MOESM2_ESM.docx]

**Table S2**: Significant determinants of unintended pregnancy reported from each study.

| **First author (publication year)** | **Statistically significant variables with AOR(95%CI)** |
| --- | --- |
| **Adenager et al (2017)** | **Distance to health facility**  >2.5 km: **1.6** (**1**.**1**, **2**.**5**)  ≤2.5 km: Ref |
| Alema et al (2019) | **Occupation Knowledge**  Farmer: 3.38 (1.35, 8.49) Not knowledgeable: 1.49 (1.142,2.262  Student: Ref Knowledgeable: Ref.  **Health facility visited first Sought treatment from TORH before visiting HF**  Public health center: 0.42 (0.25, 0.70) Yes: 1.71 (1.07, 2.73)  Public hospital: Ref. No: Ref.  **Financial problem** **Severely ill with the diseases**  Yes: 2.26 (1.26, 4.06) Yes: 2.09 (1.10, 3.98)  No: Ref. No: Ref. |
| Asefa et a l(2014) | NR |
| Asres et al (2017) | **Age Occupation Knowledge**  30-44: 8.74 (4.71, 16.23) Government employee: Ref. poor: 2.79 (1.74,4.92)  ≥45: Ref. Merchant: 4.51 (1.41, 14.44) Good: Ref.  Farmer: 4.18 (1.44,12.11) |
| Asres et al (2019) | **Age:** 1.01(1.001,1.03) **First action to illness Knowledge towards TB**  **Type of TB** Self-treatment: 1.72(1.07,2.75) Good: 0.67(0.46,0.98)  EXPTB: Ref. Consult HCP: Ref. Poor: Ref.  Pulmonary positive: 1.54(1.03,2.29)  **First visited HCF Travel time to first HCF**  Health center: 0.25(0.07,0.94) >1 h: 1.37(1.01,1.88  Hospital: 0.17(0.05, 0.64) <=1 h: Ref.  Private clinic: 0.22(0.06,0.81)  Health post: Ref |
| Belay et al (2012) | **Self-treatment** **First health action**  No: Ref. Formal health provider: Ref.  Yes: 3.99 (1.50,10.59) Non-formal provider: 6.18 (1.84, 0.76) |
| Bogale et al (2017) | **House hold income** **Rural residency** **HIV positive status** **seeking care from informal care providers**  −0.006 (−0.008, −0.004) 8.00 (5.26, 10.75) −8.97 (−12.02, −5.94 8.09 (5.50, 10.69) |
| Demissie et al (2002) | No statistically significant variable found |
| Fuge et al (2018) | **Occupation Residence** **knowledge**  **Distance from HF**  Unemployed : 10.08 (4.85–20.69) Urban: 2.36 (1.64,3.40) Poor: 0.44 (0.23,0.84) >5 km: 1.57 (1.03,2.41)  Farmer: 13.46 (9.15–19.85) Rural: Ref. Good: Ref. ≤ 5 km: Ref.  Private worker: 7.38 (4.31–12.77) **Monthly income**  Student: 16.69 (9.54–29.15) <100 EB: 3.38 (2.01–5.66)  House wife: 16.77 (11.15–25.31) 100–500 EB: 1.92 (1.00–3.73)  Daily laborer: 7.69 (1.54–38.08) >500 EB: Ref.  Government employee: Ref. |
| Gebeyehu et al (2014) | **Education level** **Place of residence**  Illiterate: 3.73 (1.87, 7.44) Rural: 1.74 (1.04, 2.90)  Read and write: 2.58 (1.08, 6.15) Urban: Ref.  Elementary: 3.39 (1.57, 7.33)  Junior/secondary: 2.74 (1.36, 5.49)  College and above: Ref. |
| Gebreegziabher (2016) | **First health care seeking action**  **Age** **knowledge about TB**  Visited non-formal health provider: 47.56 (26.31, 85.99 15–24:Ref. Good: Ref.  Self-treatment using various remedies at their homes: 10.11 (4.53, 22.56) ≥ 45: 2.99 (1.14, 7.81) Poor: 2.33 (1.34–4.05)  Visited formal health provider :Ref. |
| Getnet et al (2019) | **Residence Livelihood** **Walking distance to nearest HF**  Rural: 2.1 (1.3, 3.7) Pastoralism: 2.1 (1.2, 3.6) More than an hour: 3.2 (1.9,5.6)  Urban: Ref. Non-pastoralism: Ref. Within an hour: Ref.  **Knowledge of main TB symptoms: Hoped illness go- away gradually Severity of disease**  Poor: 2.7 (1.5, 4.8) Yes: 2.1 (1.3, 3.5) Mild: 1.6 (1.01,2.6)  Satisfactory: Ref. No: Ref. Moderate/severe: Ref. |
| Hussen et al (2012) | **Residence** **Place of first visit** **Educational status**  Rural : 5.1(1.39,18.90) TH, Drug shops, PC and HP: 12.2(2.80,53.11) illiterate: 2.7(1.22,9.73)  Urban: Ref. Health Center and hospital: Ref. Literate: Ref.  **Level of severity before first visit to HF** **Distance from the HF to home** **Sold their assets to go HF before first visit**  Working sometimes: 15.1(2.84,80.37) >10 Km: 2.5(1.01,9.44) yes: 6.67(3.81,11.99)  Bedridden: Ref. ≤10 Km: Ref. No: Ref. |
| Mekonnen et al (2014) | **First action to illness**  **Education** **TB category** **Distance**  No prior treatment: Ref. Collage & above: Ref. PTB+:Ref. ≤10 km: Ref.  Holy water: 10.6 (3.9,27.8) Illiterate: 6.18(1.34,28.43) Extra-PTB: 10.12(3.07,33.37) >10 km: 3.15(1.17,10.8)  Traditional healer: 4.29 (1.13,16.37)  private drug store: 6.8 (2.5,18.78) |
| Mesfin et al (2015) | **Formal schooling** **Patient suspected TB** **Actions taken before**  **Thought the illness not serious**  No: 2.46 (1.21,5.01) Yes: 2.5 (1.18,5.29) Treated: 2.9 (1.42,6.08) Yes: 2.39 (1.52,3.78)  Yes: Ref. No: Ref. Did nothing: Ref. No: Ref. |
| Seid et al (2018) | **TB category**  SPPTB: Ref.  SNPTB: 2.3 (1.25,4.21)  EPTB: 2.3 (1.28,4.07) |
| Shiferaw et al (2019) | **Type of TB** **Treatment history of patients** **Heard information about TB**  Pulmonary: Ref. New: 2.94 (1.26–6.84 Yes: Ref.  Extra pulmonary: 3.41(1.46, 7.95) Previously treated: Ref. No: 3.37 (1.43,8.00) |
| Tsegaye et al (2016) | **Informal treatment sources**  **Knowledge** **Residence**  Traditional healers: 6.80(3.62, 12.79) Good: Ref. rural: 1.59(1.03,2.45)  Holly water: 2.58(1.61, 4.15) Poor: 1.87(1.24, 2.83) urban: Ref.  Drug store: 2.69(1.55,4.66)  Health institution: Ref. |
| Wondimu et al (2007) | **Gender**  **Residence** **Educational status** **Family income**  Male: Ref. Urban: 1.46(1.1, 1.95) No education: Ref. 151 – 220: 1.797 (1.10, 2.94)  Female: 0.63 (0.47, 0.84) rural: Ref. 9-12: 2.07(1.04, 2.41) ≤70 Birr: Ref. |
| Yarlagadda et al (2018) | NR |
| Yimer et al (2005) | **Age** **Residence** **Health provider visit** **Self-treatment**  15-24:Ref. >10km: Ref. Formal: Ref. Yes: 1.69 (1.04, 2.75)  >45: 2.62 (1.13, 6.09) ≤10 km: 3.81 (2.21, 6.57) non-formal: 0.34 (0.20, 0.57) No: Ref.  **Knowledge of TB**  Low: 1.89 (1.15, 3.10)  High: Ref. |
| Yimer et al (2014) | **Residence** **Type of TB**  Urban: Ref Pulmonary: Ref.  Rural: 3.4 (1.3, 8.9) Extra pulmonary: 2.6 (1.3, 5.4) |
| Yirgu et al (2017) | **Age** **Mode of transportation** **Previous TB treatment**  15-24: Ref. Traditional or modern: Ref. Yes: Ref.  25-34: 0.81(0.44,1.47) On foot: 2.62(1.25,5.49) No: 16.16(9.94,26.26)  35-44: 0.53(0.28,1.01)  45-54: 0.31(0.15,0.61) |
| Zeleke et al (2014) | **Place of residence** **Monthly income in ETB**  Rural: 3.49(1.20-10.10) 1-450 ETB : 4.96(1.71-14.35)  Urban: Ref. No regular income: Ref. |

*AOR=adjusted odds ratio, CI=confidence interval, HF=health facility, Ref. =reference, TR=traditional healers, HP=health post, TORH=Traditional or religious healers, PC= private clinic, ETB =Ethiopian birr, NR=not reported*
